# ASIC1a Inhibitor mambalgin-2 Suppresses the Growth of Leukemia Cells by Cell Cycle Arrest

**DOI:** 10.32607/actanaturae.10949

**Published:** 2020

**Authors:** M. L. Bychkov, M. A. Shulepko, V. Y. Vasileva, A. V. Sudarikova, M. P. Kirpichnikov, E. N. Lyukmanova

**Affiliations:** Shemyakin-Ovchinnikov Institute of Bioorganic Chemistry, Russian Academy of Sciences, Moscow, 117997 Russia; Institute of Cytology RAS, St-Petersburg, 194064 Russia; Biological Faculty of Lomonosov Moscow State University, Moscow, 119992 Russia

**Keywords:** chronic myelogenous leukemia, acid-sensing ion channels, three-finger proteins, cell cycle, Ly6/uPAR

## Abstract

Although tyrosine kinase inhibitors have brought significant success in the treatment of chronic myelogenous leukemia, the search for novel molecular targets for the treatment of this disease remains relevant. Earlier, expression of acid-sensing ion channels, ASIC1a, was demonstrated in the chronic myelogenous leukemia K562 cells. Three-finger toxins from the black mamba (*Dendroaspis polylepis*) venom, mambalgins, have been shown to efficiently inhibit homo- and heteromeric channels containing the ASIC1a subunit; however, their use as possible antitumor agents had not been examined. In this work, using the patch-clamp technique, we detected, for the first time, an activation of ASIC1a channels in the leukemia K562 cells in response to an extracellular pH decrease. Recombinant mambalgin-2 was shown to inhibit ASIC1a activity and suppress the proliferation of the K562 cells with a half-maximal effective concentration (EC_50_) ~ 0.2 μM. Maximum mambalgin-2 inhibitory effect is achieved after 72 h of incubation with cells and when the pH of the cell medium reaches ~ 6.6. In the K562 cells, mambalgin-2 caused arrest of the cell cycle in the G1 phase and reduced the phosphorylation of G1 cell cycle phase regulators: cyclin D1 and cyclin-dependent kinase CDK4, without affecting the activity of CDK6 kinase. Thus, recombinant mambalgin-2 can be considered a prototype of a new type of drugs for the treatment of chronic myelogenous leukemia.

## INTRODUCTION

Chronic myelogenous leukemia (CML) is the most common type of leukemia in adults. Although significant progress in the treatment of CML has been made after the development of tyrosine kinase inhibitors, CML cells often prove drug-resistant, and disease progression gives rise to a pool of rapidly growing and drug-resistant cancer stem cells [1]. For this reason, the search for novel molecular targets for CML treatment remains relevant in molecular oncology.

Amiloride-sensitive acid-sensing Na^+^ channels, ASIC1a, are expressed in human leukemia cells [2]. ASICs are the most sensitive molecular sensors of extracellular pH changes in mammals. Today, the six known isoforms of these channels are expressed in the membranes of neuronal and non-neuronal cells, where ASICs are involved in such essential regulatory functions as synaptic plasticity, learning, memory, nociception, as well as the development of various pathologies [3]. However, the functional activity of ASICs in leukemia cells has not been studied yet. ASICs inhibition in leukemia cells can become a promising strategy in CML treatment.

**Table:** Primers used for real-time PCR

Gene	Primer		Amplicon, bp
	Forward	Reverse	
β-actin	CATGTACGTTGCTATCCAGGC	CTCCTTAATGTCACGCACGAT	88
GPDH	ACAACTTTGGTATCGTGGAAGG	GCCATCACGCCACAGTTTC	73
RPL13a	TCAAAGCCTTCGCTAGTCTCC	GGCTCTTTTTGCCCGTATGC	104
ASIC1a	CGAAGCAGGCATCAAAGTGC	TTTGGATGATAGGGAGCCACG	642
ASIC2	CACCAAGACTTCACCACAGTGTTT	TGTAGCGGGTCTCACAGTCA	409
ASIC3	TACAAGAACTGTGCCCACCC	GGTCTTCGGAACAGAGCAGA	502
ASIC4	GAGGAGAGAGACAAGCGGCA	GTCCAGCATGATCTCCAGGC	930

Mambalgins were isolated from the black mamba (*Dendroaspis polylepis*) venom [4]. Mambalgins exhibit a strong analgesic activity and efficiently inhibit the ASIC1a channels [4, 5]. Here, we studied the effect of recombinant mambalgin-2 on model CML K562 cells. For the first time, currents mediated by the acid-sensing ion channels ASIC1a were detected in the K562 cells. Mambalgin-2 was shown to inhibit the activity of these channels and K562 cell proliferation by inducing cell cycle arrest in the G1 phase and suppressing an activation of cyclin D1 and cyclin-dependent kinase CDK4. Therefore, recombinant mambalgin-2 can be considered a prototype of novel drugs for the targeted therapy of human chronic myelogenous leukemia.

## EXPERIMENTAL

Human chronic myelogenous leukemia K562 cells (Russian Cell Culture Collection, Institute of Cytology, Russian Academy of Sciences) were cultured in a RPMI-1640 medium (PanEco, Russia) supplemented with 10% fetal bovine serum (Hyclone, UK) at 37°C in the presence of 5% CO_2_. For electrophysiological experiments, the cells were seeded onto coverslips (4 × 4 mm) pre-coated with poly-*DL*-lysine (Sigma Aldrich, USA).

The level of ASIC mRNA expression in the K562 cells was quantified by real-time PCR. Total RNA was extracted from the K562 cells using the ExtractRNA reagent (Evrogen, Russia), treated with DNase I (Sigma Aldrich), and purified using the CleanRNA Standard kit (Evrogen). cDNA was synthesized using Mint reverse transcriptase (Evrogen); PCR was conducted with the ready-to-use SYBRGreen HS mix (Evrogen) and primers (*Table*) on a Roche LightCycler 96 amplifier (Roche, Switzerland). The expression level of the target genes was normalized to the expression level of the *β-ACTIN, GPDH*, and *RPL13a *housekeeping genes using the LightCycler SW software (Roche).

The transmembrane ion currents were detected using whole-cell configuration of the patch-clamp technique. The high-precision patch-clamp amplifier Axopatch 200B and digitizer Digidata 1550A (Molecular Devices Corp., USA) were used. Pipettes with a resistance of 3–6 MΩ were made from standard BF 150-110-10 filaments on a P-97 pipette puller (Sutter Instrument, USA). The extracellular solution in the chamber contained 145 mM NaCl, 2 mM CaCl_2_, 1 mM MgCl_2_, and 10 mM HEPES/TrisOH (pH 7.4); the intracellular pipette solution contained 140 mM K-aspartate, 5 mM NaCl, 2 mM EGTA, 1 mM MgCl_2_, 20 mM HEPES/TrisOH, and 0.176 mM CaCl_2_ to maintain the free calcium ion concentration at 0.01 μM (pCa = 8). The extracellular solution with pH 5.0 contained MES. The currents were recorded at a membrane potential of –50 mV. The experimental data were analyzed using the pCLAMP 10.6 software (Molecular Devices Corp.).

Mambalgin-2 and its mutant variant, L32A, were produced in *Escherichia coli *as previously described [6]. In order to determine the cell doubling time, the K562 cells were seeded into 96-well cell culture plates (5 × 10^5^ cells/mL); the medium was replaced every 24 h during the cultivation. Cell count at different time points was determined using the Goryaev chamber (MiniMed, Russia) after staining with trypan blue (PanEco). To study the effect of mambalgin-2 on proliferation, the K562 cells were seeded into 96-well culture plates (5 × 10^5^ cells/mL or 5 × 10^4^ cells/well); mambalgin- 2 (from the 2 mM stock solution in 100% dimethyl sulfoxide (DMSO)) was dissolved in the culture medium and added to the cells at different concentrations. The cells were then incubated for 72 h, with medium replacement every 24 h (prior to medium replacement, the cells were sedimented at 200*g *for 5 min). The maximum DMSO concentration did not exceed 0.5%; added DMSO had no effect on cell growth. Cell proliferation was evaluated using an MTT assay. MTT was added to the cells to a final concentration of 0.1 mg/mL, and the cells were incubated for 4 h. The resulting formazan crystals were dissolved in isopropanol, supplemented with 75 mM HCl. The optical density in the plate wells was determined on a Bio-Rad 680 plate reader at 540 nm, with background subtraction at 655 nm. The optical density in the plate wells was normalized to the optical density of the wells containing untreated cells and analyzed using the Graphpad Prism 6.0 software (GraphPad Software, USA).

To analyze the influence of mambalgin-2 on a cell cycle, the K562 cells were seeded into 6-well culture plates (25 × 10^4^ cells/well) and incubated with 1 μM mambalgin-2 for 72 h; the medium was replaced every 24 h. The cells were then fixed in 70% ethanol for 12 h (–20°C), washed with Earle’s solution twice, and incubated in a DNA extraction buffer (200 mM Na_2_HPO_4_ supplemented with 0.004% Triton X-100, pH 7.8) for 5 min. The cells were then washed, resuspended in Earle’s solution containing 50 mg/mL propidium iodide and 0.2 mg/mL RNase A, and analyzed on a FACSCalibur flow cytometer (Becton Dickinson, USA). The percentage of cells in each cell cycle phase was determined using the ModFit LT software (Verity Software, USA).

The effect of mambalgin-2 on the phosphorylation of the cell cycle regulators cyclin D1, cyclin-dependent kinases CDK4 and CDK6 was analyzed by Western blotting. The cells were lysed in a RIPA buffer supplemented with SIGMAFAST protease inhibitors (Sigma Aldrich). The lysates were submitted to electrophoresis in polyacrylamide gel (PAGE) and transferred onto nitrocellulose membranes (Santa Cruz, USA). The membranes were blocked in 5% skimmed milk (Dia-M, Russia) for 2 h and incubated with primary rabbit antibodies to cyclin D1 (pSer90, Antibodies online, Germany, ABIN6271254), cyclin-dependent kinase CDK4 (pThr172, Antibodies online, ABIN6271182), and cyclin-dependent kinase CDK6 (pTyr24, Antibodies online, ABIN319289), as well as mouse primary anti- β-actin antibodies (R&D Systems, USA, MAB8929) for 16 h (4°C). The membranes were then washed three times with TBS + 0.1% Tween 20 and incubated with HRP-conjugated anti-rabbit (Jackson Immunoresearch, UK, 111-035-003) or anti-mouse (Jackson Immunoresearch, 715-035-150) secondary antibodies. Next, the membranes were washed with TBS + 0.1% Tween 20 four times and protein bands were detected using an ECL substrate (Bio-Rad, USA) on an LAS500 chemidocumenter (GE Healthcare, USA). Band intensity was quantified using the ImageJ software (NIH, USA).

## RESULTS AND DISCUSSION

**Fig. 1:**
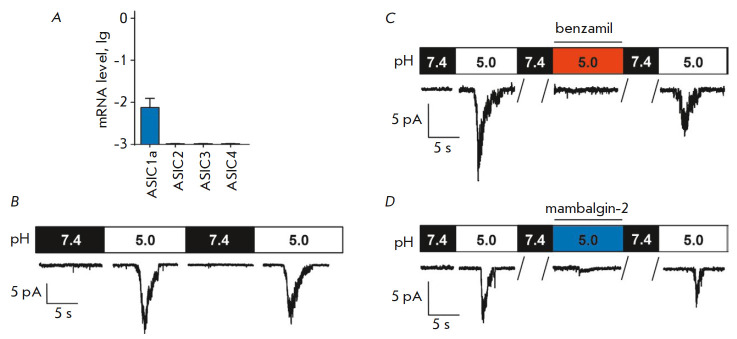
The effect of mambalgin-2 on the acid-sensing sodium channels in the K562 cells. *A *– Analysis of the mRNA expression of various ASIC types using real-time PCR (n = 3). *B *– Activation of the channels caused by rapid changes in the extracellular solution pH from 7.4 to 5.0; membrane voltage was clamped at –50 mV. *C *– Currents activated by pH 5.0, before, during, and after 1 μM benzamil addition to the extracellular solution. *D *– Currents activated by pH 5.0, before, during, and after 1 μM mambalgin-2 addition to the extracellular solution

It was shown earlier that ASIC1a channels are expressed in human leukemia K562 cells [2]. In this study, we confirmed by real-time PCR that *ASIC1a *is expressed in the K562 cells, while no expression of *ASIC2*,* ASIC3, *or *ASIC4 *was detected (*Fig. 1A*). An extracellular medium pH decrease is known to activate the acid-sensing ion channels, mediating tumor cell proliferation and migration [7]. In order to determine whether the decrease in extracellular pH activates inward currents through the membranes of the K562 cells, we carried out electrophysiological experiments using whole-cell configuration of the patch-clamp technique. We established that rapid change in the extracellular solution pH from 7.4 to 5.0 leads to an activation of acid-sensing Na^+^ channels (*Fig. 1B*). After pH was reduced, the current amplitude rapidly increased up to maximum values and then declined at a slower rate during desensitization to the steady-state level, which is typical of the acid-sensing ion channels ASIC1a [8]. The current amplitudes at pH 5.0 were 7–16 pA (*n* = 6). Inward currents at low pH (5.0) were blocked by 1 μM benzamil, an amiloride derivative, thus proving that the pH-sensing ion channels in the K652 cells belong to the ASIC1a family (*Fig. 1C*). Hence, the ASIC1a-mediated transient currents caused by the acidification of the environment are typical of K562 cells. Mambalgin-2 (1 μM), added to the extracellular solution (pH 5.0), completely inhibited the activation of the acid-sensing Na^+^ channels in the K562 cells (*Fig. 1D*). Subsequent replacement of the extracellular solution with a similar one containing no mambalgin-2 (pH 7.4 and 5.0) reactivated the acid-sensing ion channels (*Fig. 1D*), thus indicating that the effect of mambalgin-2 is reversible.

**Fig. 2:**
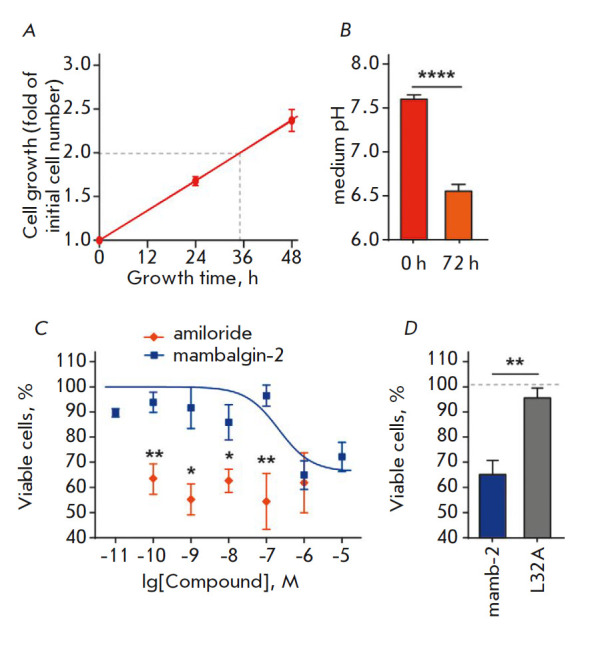
The effect of mambalgin-2 on the growth of the K562 cells. *A *– K562 cell growth without any substances, with medium change every day. The cell doubling time was calculated using linear regression. *B *– Changes in the medium pH upon incubation of cells for 72 h. Data are presented as pH ± SEM (n = 3); **** (p < 0.0001) indicates a significant difference between groups of data according to the two-tailed t-test. *C *– The effect of different mambalgin-2 concentrations on cell proliferation. Data are presented as % of the control (untreated cells) ± SEM (n = 3–6). * (p < 0.05) and ** (p < 0.01) indicate a significant difference between groups of data according to the two-tailed t-test. *D *– The antiproliferative activity of mambalgin-2 and its mutant variant, L32A. The data are presented as % of the control (untreated cells, dashed line) ± SEM (n = 3); ** (p < 0.01) indicates a significant difference between groups of data according to the two-tailed t-test

The doubling time for the K562 cells determined by cell counting after trypan blue staining was ~ 35 h (*Fig. 2A*). We tested pH of the cell medium during the cultivation of the K562 cells with daily medium replacement and found that the K562 cells cause its acidification after 72 h and reduce pH from 7.6 ± 0.06 to 6.6 ± 0.07 (*Fig. 2B*), which is sufficient for the activation of ASIC1a, one of the most sensitive to the pH drop acid-sensing ion channel [3]. Therefore, we assume that active metabolism and rapid proliferation of the K562 cells result in an acidification of the culture medium, thus activating acid-sensing ion channels.

It was shown earlier that ASIC1a inhibition by psalmotoxin (PcTx1) from the tarantula (*Psalmopoeus cambridgei*) venom or benzamil down-regulates tumor cell growth [9]; therefore, we assessed the effect of mambalgin-2 on the growth of the K562 cells. A MTT assay demonstrated that incubation of the K562 cells with mambalgin-2 for 72 h reduces the percentage of viable cells to 68.2 ± 5.8% compared to untreated cells (control), with a half-maximal effective concentration (EC_50_) of 179.9 ± 20.8 nM. The antiproliferative effect of mambalgin-2 in a concentration range varying from 10-10 to 10-7 M was much weaker compared to that of amiloride, the ASIC1a inhibitor (*Fig. 2C*). In order to confirm that the antiproliferative activity of mambalgin- 2 is associated with its interaction with ASIC1a, we used the mutant variant of the toxin carrying the L32A mutation, which exhibits low affinity to ASIC1a and reduced the inhibitory activity towards ASIC1a [10]. Indeed, unlike recombinant mambalgin-2, the L32A mutant variant had no effect on the growth of the K562 cells (*Fig. 2D*). Therefore, ASIC1a appears to be the main molecular target of mambalgin-2 in the K562 cells. Evidently, proliferation of the K562 cells causes acidification of the medium, resulting in an activation and subsequent desensitization of ASIC1a channels, while mambalgin-2 inhibits the growth of the K562 cells by maintaining the channels in a desensitized state. These findings are consistent with the earlier data on the interaction between mambalgin-2 and the ASIC1a channel in a desensitized state [4, 11].

**Fig. 3:**
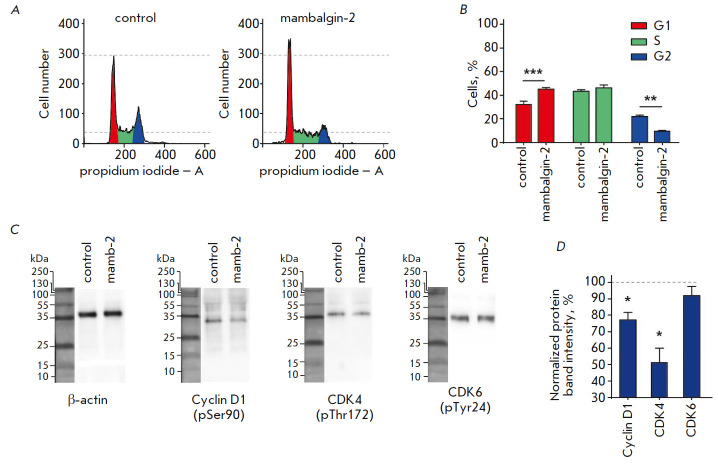
The effect of mambalgin-2 on the cell cycle in the K562 cells. *A *– Representative histogtamms of cell nuclei population distribution after 72 h incubation in absence (control) or presence of mambalgin-2. *B *– % of cells in each cell cycle phase. The data are presented as % of cells in each cell cycle phase ± SEM (n = 4); ** (p < 0.01) and *** (p < 0.001) indicate the significant difference between the control (untreated cells) and mambalgin-2-treated cells according to the two-tailed t-test. *C *– Representative Western blot showing the influence of mambalgin-2 on the phosphorylation of the cell cycle regulators. *D *– Quantification of the band intensities of cell cycle regulators after the cells were incubated with mambalgin-2. Data are presented as normalized to the β-actin band intensity, where untreated cells are taken as the control (100%, dashed line) ± SEM (n = 4); * (p < 0.05) indicates the difference between the control (untreated cells) and the cells treated with mambalgin-2 according to the two-tailed t-test

PcTx1 and benzamil were previously found to induce cell cycle arrest in the G0/G1 phase and inhibit cyclin-dependent kinases [9]. Incubation of the K562 cells in the presence of mambalgin-2 also increased the cell number in the G1 phase by 33% and reduced the number of cells in the G2 phase by 54%, pointing to a G1-phase cell cycle arrest (*Fig. 3 A,B*). It was shown by Western blot analysis that incubation of the K562 cells with mambalgin-2 inhibits the phosphorylation of cyclin D1 and cyclin-dependent kinase CDK4 but not cyclin-dependent kinase CDK6 (*Fig. 3 C,D*). A complex formation between cyclin D1 and cyclin-dependent kinase CDK4 is required for CDK4 activation and cell cycle progression into the S phase. Therefore, the inhibition of cyclin D1 and CDK4 activation leads to cell cycle arrest. Upregulation of cyclin D1 in the acceleration phase of CML is considered a negative prognostic factor. So, cyclin D1 inhibition can become a new strategy in the treatment of chronic myelogenous leukemia [12].

## CONCLUSIONS

Functionally active acid-sensing ion channels ASIC1a were detected for the first time in leukemia K562 cells. Mambalgin-2 was shown to suppress the activity of these channels and to inhibit leukemia cell proliferation by causing G1-phase cell cycle arrest and reducing the activity of the cell cycle regulators cyclin D1 and cyclin- dependent kinase CDK4. These findings indicate that ASIC1a is a potential molecular therapeutic target for the treatment of CML, while recombinant mambalgin- 2 can be considered a prototype of novel drugs for targeted anti-cancer therapy.

## Abbreviations

DMSO: dimethyl sulfoxide
ASICs: acid-sensing ion channels
CDK: cyclin-dependent kinase
CML: chronic myelogenous leukemia
MTT: 3-(4,5-dimethylthiazol-2-yl)-2,5-diphenyl-tetrazolium bromide
PcTx: psalmotoxin
TBS: Tris-buffered saline
